# Case management of mpox: Where we are and where we desire to be

**DOI:** 10.4102/jphia.v16i1.1228

**Published:** 2025-03-31

**Authors:** Solomon F. Woldetsadik, Ebenezer O. Daniel, John Masina, Joseph C. Okeibunor, Samuel Boland, Hilary K. Njenge, Nicaise Ndembi, Ngashi Ngongo, Otim P.C. Ramadan, Fiona Braka, Abdou S. Gueye

**Affiliations:** 1 Regional Office for Africa, Emergency Preparedness and Response Hub, World Health Organization, Nairobi, Kenya; 2 Regional Office for Africa, Emergency Preparedness and Response Cluster, World Health Organization, Brazzaville, Democratic Republic of Congo; 3 Africa Centre for Disease Control, Addis Ababa, Ethiopia

## Introduction

The resurgence of mpox and its declaration as a public health emergency of international concern (PHEIC) by the World Health Organization (WHO) twice in the last 2 years is alarming and demands attention.^1,2^ This situation poses significant challenges and opportunities for improving clinical case management. Urgent collaborative efforts are needed to develop refined clinical guidelines, targeted pharmacological approaches, and effective preventive measures. Innovative approaches are particularly crucial in the WHO African region, where outbreaks of infectious diseases such as mpox further strain the already-fragile health systems.^3,4^

## Current approach to case management and gaps

Mpox case management currently draws on experiences from smallpox treatment, as both viruses belong to the orthopoxvirus family.^5,6^ Antiviral drugs such as brincidofovir and tecovirimat show promise, with tecovirimat recommended for severe cases under WHO Monitored Emergency Use of Unregistered and Experimental Interventions (MEURI).^7^ Vaccination of at-risk populations is another promising strategy, but vaccine distribution has been slow, especially in the WHO African region because of limited supply and local manufacturing challenges.^8^ Further clinical evidence is required to validate the effectiveness of these therapeutics and vaccines, which remain largely inaccessible in the African region.

The current approach, involving isolation protocols, symptom treatment, and supportive care, falls short in achieving desired outcomes in both endemic and non-endemic regions.^9^ Treatment efficacy varies because of differences in clinical presentations, ranging from mild to severe cases requiring multidisciplinary management.^10^ Symptoms include fever, headaches, pustular rash, lymphadenopathy, and muscle aches. Management focuses on pain relief, hydration, nutritional support, and antiviral therapy for high-risk patients, such as immunocompromised individuals.^11^

In response to the mpox epidemic in the WHO African region, public health stakeholders have introduced interventions to improve case management outcomes. Training clinicians in triage, isolation, and case management principles have been prioritised, with nearly 5000 health workers trained. This capacity building has documented evidence in reducing mortality rates in healthcare facilities.^12^

In hotspot areas, such as the Democratic Republic of Congo (DRC) and Burundi, treatment units have been mapped and assessed. Infection prevention and control (IPC) assessments revealed critical gaps in screening (37%), isolation (27%), and hand hygiene (37%). In Kinshasa, hand hygiene compliance was as low as 6%. Similar gaps were found in Uganda. These assessments have informed interventions, including health worker training on IPC protocols and the provision of personal protective equipment (PPE), which is vital for preventing nosocomial infections in healthcare settings.^13^

Efforts to improve clinical data collection include training over 200 users on the mpox global clinical data platform. Furthermore, an essential medicines list for mpox management has been distributed to member states. A nutrition package developed with the World Food Programme (WFP) has been implemented in high-admission facilities in the DRC and Burundi, improving treatment outcomes and reducing case fatality rates as exemplified in a similar scenario laced with uncertainties.^14^

Despite these efforts, critical gaps remain. Limited knowledge about mpox transmission dynamics, including the role of asymptomatic carriers, zoonotic reservoirs, and environmental factors, needs further research. Clinical manifestations and complications require expert collaboration to refine case classification criteria for more precise treatment. Limited access to antivirals and vaccines hampers healthcare systems, particularly in resource-limited settings with high mpox prevalence, such as the conflict-affected regions in eastern DRC.

## Future directions in mpox management

The future of mpox management must address current gaps while prioritising inclusivity and accessibility. Enhanced surveillance in the WHO African region is essential, including real-time data sharing, strategic contact tracing, and advanced molecular diagnostic techniques to quickly identify and prevent outbreaks. Integrating mpox into the One Health framework can help track zoonotic transmission and enable targeted public health responses.^15^

Scaling up clinical trials and research across all regions is critical. Efforts should evaluate the safety and efficacy of vaccines and antivirals, particularly tecovirimat. Research into novel antivirals especially for immunocompromised patients prone to severe disease outcome,^16^ reinfection risks, and vaccine efficacy duration is also necessary to refine public health policies. Addressing disparities in access to therapeutics and vaccines is essential for improving treatment outcomes. International collaboration can enable pooled procurement, subsidised distribution, and innovative delivery systems such as mobile vaccination units to increase coverage in remote areas.^17^

Developing specialised clinical guidelines, led by WHO and Africa Centre for Disease Control and Prevention (Africa CDC) with input from technical working groups, is vital. These guidelines should include protocols for managing severe and immunocompromised patients. Health worker training on these protocols will ensure effective application, improving patient outcomes.^18^

In conclusion, the surge in mpox cases since 2022 highlights vulnerabilities in global and regional healthcare systems, emphasising the need for improved case management. A more holistic approach, including research investment, increased accessibility, and updated guidelines, is essential. By adopting a globally inclusive strategy, the transmission and resurgence of mpox can be curtailed. The time to act is now!
